# Real-time quantification of wild-type contaminants in glyphosate tolerant soybean

**DOI:** 10.1186/1472-6750-9-16

**Published:** 2009-03-06

**Authors:** Elena Battistini, Enrico Noli

**Affiliations:** 1 Seed Research and Testing Laboratory (LaRAS), Department of Agroenvironmental Sciences and Technologies (DiSTA), Alma Mater Studiorum University of Bologna, viale Fanin 40, Bologna 40127, Italy

## Abstract

**Background:**

Trait purity is a key factor for the successful utilization of biotech varieties and is currently assessed by analysis of individual seeds or plants. Here we propose a novel PCR-based approach to test trait purity that can be applied to bulk samples. To this aim the insertion site of a transgene is characterized and the corresponding sequence of the wild-type (wt) allele is used as diagnostic target for amplification. As a demonstration, we developed a real-time quantitative PCR method to test purity of glyphosate tolerant (Roundup Ready^®^, RR) soybean.

**Results:**

The soybean wt sequence at the RR locus was characterized and found to be highly conserved among conventional genotypes, thus allowing the detection of possibly any soybean non-trait contaminant. On the other hand, no amplification product was obtained from RR soybean varieties, indicating that the wt sequence is single copy and represents a suitable marker of conventional soybean presence. In addition, results obtained from the analysis of wt-spiked RR samples demonstrate that it is possible to use the real-time PCR assay to quantify the non-trait contamination with an acceptable degree of accuracy.

**Conclusion:**

In principle this approach could be successfully applied to any transgenic event, provided that the wild-type sequence is conserved and single copy. The main advantages of the assay here described derive from its applicability to bulk samples, which would allow to increase the number of single seeds or plants forming the analytical sample, thus improving accuracy and throughput while containing costs. For these reasons this application of quantitative PCR could represent a useful tool in agricultural biotechnology.

## Background

In 2007 the cultivation of transgenic plants has reached 114 million hectares, mainly concentrated in the US, Argentina and Brasil [1]. Herbicide tolerance is the prevailing trait, with RR soybean varieties (event GTS 40-3-2) being grown on about half of the global biotech area. The use of transgenic technology, and in particular of herbicide tolerant genotypes, requires seed with high trait purity: in the US, for instance, RR soybean lots must be at least 98% pure in terms of seeds tolerant to glyphosate. For this purpose seed companies carry out extensive quality checks, usually consisting of a bioassay in which either seeds are germinated on a substrate containing the herbicide, or seedlings are sprayed with it [2]. While bioassays are the most common practice to test herbicide tolerance, immunoassays like those in form of immunostrips or ELISA are generally used for the detection of other traits, such as insect resistance [3,4].

Immunoassays and end-point PCR (epPCR), targeted to transgene-specific proteins or DNA sequences respectively, are currently widely utilized to detect the unintended, adventitious presence of genetically modified organisms (GMOs) in conventional products (hereafter indicated as AP) by analyzing bulk samples [5,6]. However, since these proteins or sequences are dominant markers they are not diagnostic of the presence of non-GM contaminants in GM materials. For this reason checking trait purity requires the analysis of individual seeds, thus raising the cost of testing, due to the laborious procedures involved and/or the use of expensive test kits.

Real-time quantitative PCR (rtqPCR) has found numerous applications in research and diagnostics, both in the animal and in the plant sciences [7-9]. For the plant biotech industry it represents a useful tool for the determination of copy number [10,11] and zygosity [12,13] of the inserted genes in the development of transgenic varieties. For regulatory purposes, this technology, in particular when associated with hydrolysis probes, is commonly utilized in the food/feed as well as in the seed sector for the detection and quantification of AP in conventional material [see Additional file 1] [14-16]. As a matter of fact, for this purpose, rtqPCR methods are not only widely used, but they are also required by the EU GMO legislation. EC/787/2004 recommends the use of DNA based quantification of the GMO content in seeds, food and feed and this has to be done by means of rtq-PCR. Methods are submitted as a part of the authorization procedure to the Community Reference Laboratory (CRL) which coordinates the validation process. Despite the great abundance of publications [5,6] and the availability of on-line databases [17,18] largely dedicated to rtqPCR methods for AP testing, up to now there were no reports on the application of rtqPCR to assess the purity of GM seedlots or of derived agricultural products. In fact, due to the exponential nature of PCR, the rtqPCR assays designed so far, targeted to the amplification of the transgenic sequence, are not suitable for checking trait purity in GM seedlots. For example, in the case of a glyphosate tolerant soybean variety, assuming that all the seeds with the trait were homozygous, only one threshold cycle (Ct) difference would actually be expected between a perfectly pure (100% herbicide tolerant) and a "very bad" (50% herbicide tolerant) seedlot [see Additional file 2].

Moreover, since seed and grain lots are made up of discrete particles, other strategies for the quantification of AP can be applied besides rtqPCR. Seed companies currently check seedlot compliance with regulations through the qualitative determination of the presence of GM contaminants on a certain number of sub-samples, usually by immunoassay or epPCR [19]. For a given quality level and a desired statistical confidence, a lot is accepted or rejected if the number of positive sub-samples is either lower or higher than a defined cutoff point [see Additional file 3]. However the detection of transgenic proteins or sequences is not suitable for assessing trait purity as all the sub-samples will be detected positive.

Here we describe the development of a PCR system for the detection and quantification of conventional impurities in GM seedlots, based on the amplification of a wt allele-specific sequence at the GM locus. As a proof of concept, we applied this approach to purity testing of RR soybean by designing epPCR and rtqPCR assays that could be applied to the analysis of bulk samples.

## Results

**Figure 1:**
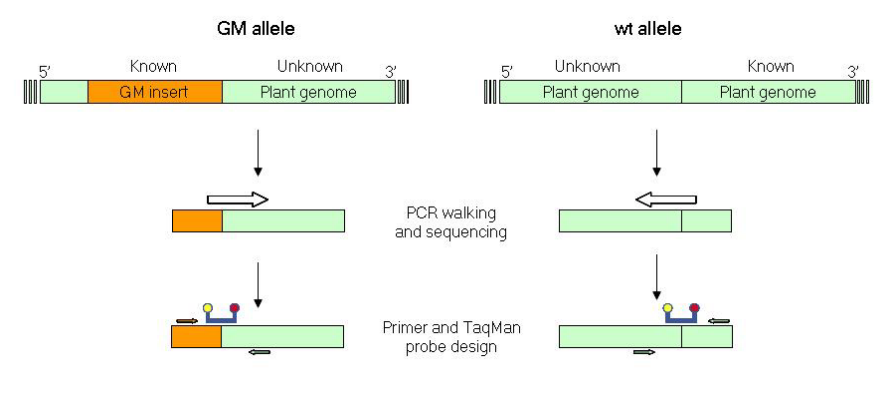
**Development of a PCR assay specific for a transgenic event or its corresponding wt sequence at the insertion site**. On the left, event-specific assay for testing AP of GMOs in conventional lots: from the known sequence of the insert a flanking plant DNA region is characterized and oligonucleotides are designed to detect the junction. On the right, wt-specific assay for testing trait purity in GM lots: from the characterized flanking region, the wt sequence allelic to the insert is obtained and oligonucleotides are designed to detect the reconstructed plant insertion site.

The rationale underlying the development of this approach is shown in Figure 1, in parallel with the usual practice for the design of event-specific assays detecting GM contaminants. Starting from the 5' or 3' sequence flanking the transgenic insert, usually already known as part of the molecular characterization of the event, the sequence of the site of insertion is obtained in a non-GM genotype using currently available strategies. In this way it is possible to reconstruct the wt sequence made up of the known flanking DNA plus the newly sequenced region and hence to design oligonucleotides targeted to the specific amplification of the non-GM allele. Similarly to the tests aimed at the quantification of AP, the wt assay could be utilized for assessing purity of GM materials, provided that the PCR target is widely conserved among non-GM genotypes and single copy.

Molecular information regarding event GTS 40-3-2 was obtained from previous work [20]. The plant genomic sequence adjacent to the 3' *nos *end of the inserted DNA was used as a starting point of a PCR-walking procedure aimed at obtaining the wt sequence at the RR locus in non-GM material [21]. As inserted DNA we considered the functional insert (enhanced CaMV 35S promoter, chloroplast transit peptide, CP4-*epsps *and *nos *terminator), an additional portion (254 base pair; bp) of the CP4-EPSPS and a fragment (534 bp) of rearranged soybean genomic DNA [22,23]. Molecular characterization of this event has shown several DNA rearrangements at the point of integration, thus originating multiple insert/plant genome junctions, each of them a potential site for wt assay design.

**Figure 2:**
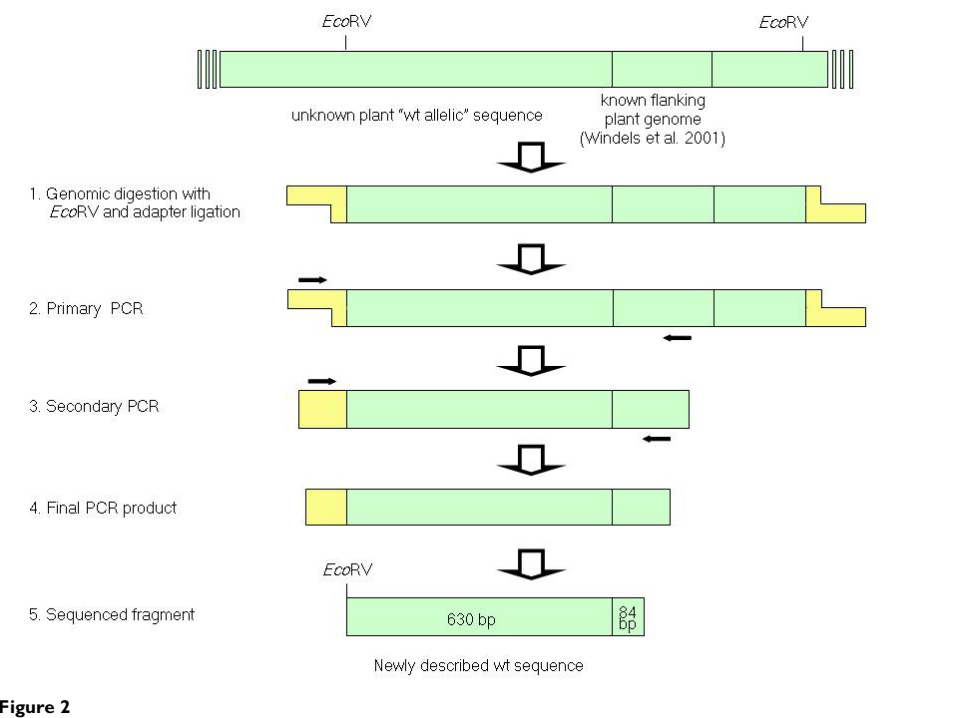
**Schematic representation of the PCR-walking approach to isolate the wt allele at the RR locus**. Top drawing: original integration site of the RR construct in conventional soybean. Steps (1–5) of PCR-walking. Only *Eco*RV fragment isolation procedure is shown.

**Figure 3:**
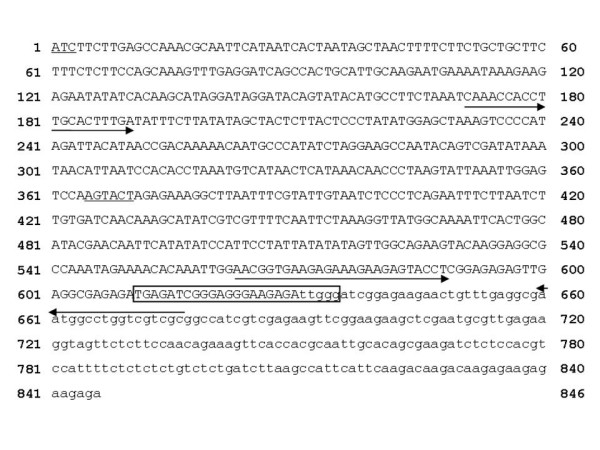
**Representation of the RR insertion site in a wild-type soybean genome**. Upper-case letters: the 630 bp sequence characteristic of wild-type obtained in this study. Lower-case letters: plant DNA sequence flanking the 3' end of the RR insert previously described [20]. Underlined letters: restriction sites for *Eco*RV and *Sca*I enzymes used in PCR-walking. Arrows and box indicate the positions of primers and probe. Forward primers for epPCR and rtqPCR start from position 171 and 563, respectively. Reverse primer was common to both assays.

Genomic DNA extracted from a conventional soybean genotype was digested with *Sca*I and *Eco*RV for which the absence of restriction sites in the 3' genomic flanking sequence had been previously ascertained (Figure 2). DNA fragments were subsequently blunt-ligated with adapters and PCR-walking reactions were performed using forward adapter primers and reverse primers specific for the known sequence flanking the 3' end of the RR insert. PCR products obtained by the nested amplification of the adapter-fragments were purified and characterized, providing sequences for the *Sca*I/plant DNA and the *Eco*RV/plant DNA. By combining the two partially overlapping sequences, a 714 bp sequence characteristic of the non-GM genome at the RR insertion site was obtained (Figure 3). Of this, 84 bp corresponded to the flanking region at the 3' end previously reported, whereas the remaining newly described 630 bp represented the wt sequence upstream of the RR insertion site [20]. However, no homology with the plant DNA flanking the 5' end of the insert was found indicating that, following the integration of the transgene, major rearrangements had taken place [20]. In addition, no homology with other sequences in GenBank was detected by BLAST analysis.

**Figure 4:**
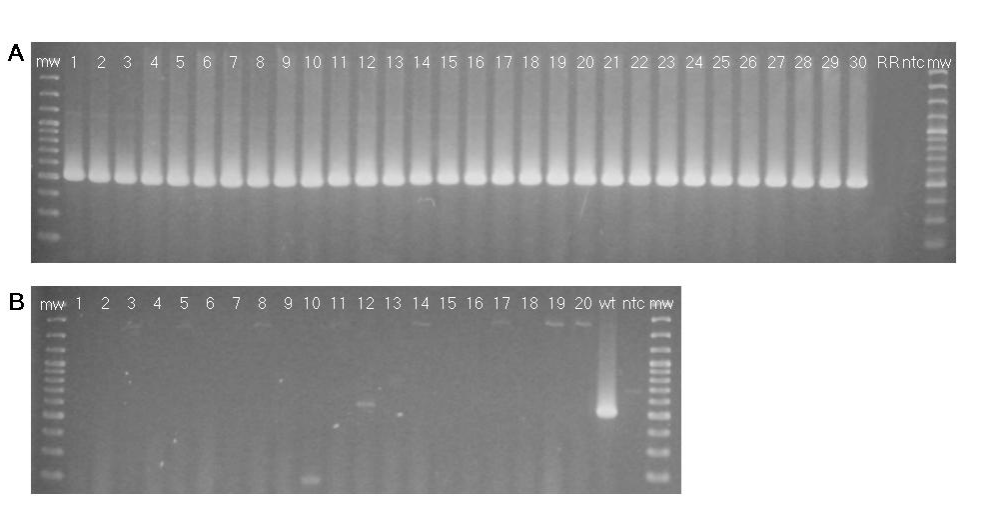
**End-point PCR detection of the 506 bp wt-specific sequence at the RR locus**. (**A**) Lanes 1–30: conventional soybean varieties. (**B**) Lanes 1–20: RR soybean varieties homozygous for the transgene. ntc = non target control. mw = molecular weight marker 100 bp ladder.

Oligonucleotides for epPCR and rtqPCR were designed on the wt allelic sequence on both sides of the insertion site, in the region corresponding to the 3' RR insert/plant genome junction (Figure 3). In order to assess the risk of false negative results, we tested the ability of the epPCR and rtqPCR systems to detect the wt sequence at the RR locus in different genotypes by analyzing 30 conventional soybean varieties of different origin (Italy, North America, China, and Japan), that represented a very wide range of germplasm [24]. The expected amplicons were obtained in all genotypes (Figure 4A), thus indicating a high degree of conservation of the wt sequence as well as the effectiveness of the assays in detecting non-trait soybean contaminants in RR seedlots. Simultaneous real-time amplification of the wt sequence and the lectin gene (*Le1*), a soybean-specific single copy target, was also performed. Interestingly, ΔCt values (Ct_*wt *_- Ct_*Le1*_) ranged from 1.13 to 1.63 (maximum ΔΔCt = 0.5), suggesting that the copy number of the wt sequence is consistent across genotypes. On the other hand, the risk of false positive results was evaluated by verifying the absence of the wt target sequence in 20 RR varieties. Since all of them turned out negative to this test (Figure 4B), we concluded that the soybean genomic region where integration has occurred is characterized by a unique, non duplicated sequence. This specificity for the wt allele makes the assay applicable to the control of the purity of any GTS 40-3-2 RR variety.

In order to assess the suitability of the real-time assay for the quantification of non-GM impurities, validation samples at different spiking levels (1.0, 2.0, 4.0, and 8.0% weight/weight) were analyzed in three different experiments. Calibration curves of ΔCt values vs log [percent wt contamination] were obtained from the analysis of standard samples representing six non-GM contents (0.1, 0.5, 1.0, 2.0, 5.0, and 10.0% weight/weight). Curves were characterized by slope values of -3.26, -3.18, and -3.34, and R^2 ^values of 0.998, 0.996, and 0.991, for experiment 1, 2 and 3 respectively. These parameters were in line with the performance requirements defined for quantitative rtqPCR methods by the European Network of GMO Laboratories [25]. Estimates of wt contamination obtained in repeated analyses (DNA extraction and PCR) of the same validation samples showed CV values ranging from 3.1 to 19.0% and were quite close to the expected values, with average absolute bias for each level lower than 25%, except for the 1% validation samples (bias 29%) (Table 1).

**Table 1:** Quantification of wt soybean percent content (weight/weight) in GTS 40-3-2 validation samples

Nominal content (%)	Sample	True content^a^ (%)	Experiment	Estimated content^b^ (%)	CV^c^ (%)	Bias^d^ (%)
1	1	1.02	1	0.75	3.1	-24
			2	0.71		-31
			3	0.75		-26
	2	1.02	1	0.75	7.5	-26
			2	0.65		-36
			3	0.73		-29
				0.72		29^e^
						
2	1	2.02	1	1.83	6.9	-10
			2	1.80		-11
			3	2.04		+1
	2	2.07	1	1.95	6.2	-6
			2	1.82		-12
			3	2.06		-1
				1.92		7
						
4	1	4.08	1	3.82	2.6	-6
			2	3.71		-9
			3	3.63		-11
	2	3.85	1	3.67	11.0	-5
			2	3.26		-15
			3	2.95		-23
				3.51		12
						
8	1	7.80	1	8.57	17.5	+10
			2	7.08		-9
			3	10.09		+29
	2	7.76	1	8.36	19.0	+8
			2	7.58		-2
			3	10.82		+39
				8.75		16

^a^Actual wt soybean content calculated from weighed non-GM and GM leaf powder; ^b^wt soybean content estimated from ΔCt analysis of samples; ^c^Coefficient of variation among the three experiments; ^d^Deviation from true value; ^e^Average absolute bias.

The absolute limit of detection (LOD), defined as the lowest concentration of wt sequence detectable in all the nine replicates, was estimated to be about 14 genome copies, corresponding approximately to 0.01% wt, while the same target was detected in only six replicates when 2.8 copies were present (Table 2). The limit of quantification (LOQ), i.e. the lowest level of wt sequence that can be reliably quantified, was assessed in three different ways. According to trueness, LOQ was determined to be 0.008%, since below this level bias showed a drastic increase. When precision was considered, LOQ was defined at 0.2%, as at lower levels variability among triplicate amplifications considerably raised. Alternatively, as in common practice, LOQ can be fixed as LOD * 3 producing the value of 0.024% (43 wt genome copies), intermediate between the previous ones. This preliminary evaluation may indicate that the method is suitable for the quantification of the wt sequence at the target concentration of 1–2%, corresponding to the level of trait purity that needs to be assessed. Therefore this assay could be utilized to quantify wt contaminations in soybean seedlots harbouring the GTS 40-3-2 event. Moreover, the same assay, used in an end-point format in conjunction with subsampling strategies, could represent a lower-technology alternative for the quantification of non-trait contaminants, analogously to the case of AP testing.

**Table 2:** Data used to determine the detection and quantification limits of the rtqPCR method for the amplification of the wt soybean sequence

Dilution level^a^	1	2	3	4	5	6	7
Expected wt DNA content (%)	5.00	1.00	0.20	0.04	0.0080	0.0016	0.0008
Estimated wt genome copies (n.)	8,889	1778	356	71	14.2	2.8	0.6
Signal rate^b^	9/9	9/9	9/9	9/9	9/9	6/9	6/9
Mean Ct value of positives^c^	26.18	28.78	31.44	33.77	35.72	35.55	35.59
Mean CV of Ct values (%)^d^	0.78	0.84	0.71	1.85	2.28	-	-
Estimated wt DNA content (%)^e^	5.00	0.82	0.13	0.026	0.0067	0.0075	0.0074
Bias (%)^f^	0	-18	-35	-35	-16	371	819

^a^Serial dilutions 1:5 of 5% wt soybean DNA in RR soybean DNA (weight/weight)

^b^proportion of PCR replicates yielding a positive signal

^c^mean of nine values

^d^CV calculated among PCR replicates

^e^based on the calibration curve passing through the point corresponding to the 5% wt with the observed Ct (26.18) and theoretical slope of -3.32

^f^deviation (%) between estimated and expected wt DNA content

## Discussion

Compared to bioassays for herbicide tolerance where phenotypic misclassifications can easily occur, particularly in distinguishing non-trait seedlings from abnormal seedlings, PCR tests provide a more objective and reliable evaluation in less time (days instead of weeks). A relevant side-benefit of the adoption of rtqPCR is that no toxic chemicals are needed in the laboratory, while special safety precautions must be taken for herbicide tolerance testing. However the main advantage of performing PCR on bulks instead of bioassays or immunoassays on single seeds or plants is that it allows to consider larger samples, thus increasing precision of estimates. Considering the standard 98% threshold of trait purity for RR soybean, the current practice of analyzing 400 individual seeds (*e.g*. by bioassay) provides estimates with a 95% confidence interval (CI) ranging from 96.1 to 99.1%, if random sampling variability is taken into account and assuming no experimental error, *i.e*. no false positive/negative results (calculated with SEEDCALC8) [26]. Under the same conditions and assuming only homozygous non-GM contaminants, it is expected that a similar precision (CI = 96.0–99.4) can be obtained with a subsampling strategy based on epPCR detection of the wt allele on 10 sub-samples of 90 seeds each. On the other hand, a considerably lower variability (95% CI = 97.5–98.5) would be expected with the rtqPCR analysis of a bulk of 3,000 seeds, just as a consequence of the increased sample size.

Among the limitations, it should be pointed out that since this approach is based on the detection of non-GM sequences in a bulk sample it can not account for the hemi- or homozygous state of individual seeds. In fact, as a consequence of the dominant inheritance of the novel trait, on one side, as well as that of the wt-marker, on the other, the level of non-GM impurities in GM material will tend to be overestimated with respect to the percentage of units that will not express the trait (homozygous wt), whereas the latter is indeed the relevant issue for commercial seed. In autogamous species where varieties are often pure lines, like in soybean, cotton, rice and wheat, there could be lots 100% herbicide tolerant but containing hemizygous individuals in variable proportions. It follows that in these species this approach could be conveniently applied whenever the non-GM contamination consisted mainly of homozygous seeds, as expected in advanced generations of selfing or when contributions from outcrossing can be assumed negligible. For the same reason these assays are not applicable to the determination of purity of GM hybrids, as maize varieties, where the trait is usually in the hemizygous state.

Like tests designed to evaluate zygosity [22,27], the assays here described, when used on single seeds or plants, can be valuable tools in the conversion of conventional varieties [28] by marker assisted backcrossing and in quality control at the early stages of seed increase, replacing laborious progeny testing. However, the rtqPCR format represents a true novelty since it provides an accurate estimate of the proportion of wt gametes that will contribute to the following generation, facilitating maintenance breeding of GM homozygous varieties and, in the case of hybrid crops, of inbred lines. Moreover it is the only possible approach for the quantification of trait purity in materials not formed of discrete particles (derived products, e.g. flour, extracts, etc.), as it could be in the next future for high value products derived from "pharma crops", or for the preparation of pure reference materials. As an obvious extension, this approach could be applied to the purity determination of any trait for which allelic discrimination is possible at a specific locus, like in the case of mutants or naturally occurring variants not strictly classifiable as GMOs.

Advantages and disadvantages of this approach compared to those of current strategies should be evaluated considering also the costs. At present, the average costs of trait purity tests by bioassays (400 seeds) or immunoassays (100 seeds) are roughly one-fifth or one-half of a rtqPCR analysis, respectively. Nonetheless, a more appropriate comparison should be conducted considering samples of the same size. Because the assessment of trait purity by rtqPCR can be conducted on bulk samples, the costs of testing are only partially affected by sample size, whereas in the case of bioassays or immunoassays they are essentially proportional to the number of seeds tested. As a consequence, rtqPCR is expected to become economically more convenient as the representativeness of the sample is increased.

## Conclusion

The cultivation of biotech plants needs high purity levels of the novel traits, in particular in the case of herbicide tolerant varieties. Current procedures for checking trait purity are applied to individual seeds or plants and require considerable time to complete. We have illustrated a new approach, based on PCR, to test genetic purity for biotech traits in seedlots and derived agricultural products that, applied to bulk samples, could potentially allow to increase the accuracy of results and lower the cost of analysis. We believe that these assay systems could be a useful tool for the breeding, maintenance and trade of transgenic varieties and hence represent a possible application in agricultural biotechnology and in particular in the seed business. A thorough comparison of the method here described with existing approaches to purity testing should be the object of further research.

## Methods

### Sample preparation and soybean DNA extraction

DNA was extracted from leaves of 30 conventional and 20 GTS 40-3-2 varieties according to the CTAB method, as described by [29]. Standard and validation samples were prepared by mixing powder obtained from GM and non-GM lyophilized leaves, checked by PCR for the presence of either the RR insert or the wt sequence to ascertain homozygosity of the relevant sequence. Levels of non-GM contamination were 0.1, 0.5, 1.0, 2.0, 5.0 and 10.0% (weight/weight) for standard samples and 1.0, 2.0, 4.0 and 8% (weight/weight) for validation samples. DNA extraction from these samples was carried out as above. DNA concentration was estimated spectrophotometrically and the samples were all diluted in sterile H_2_O to the working solution of 40 ng/μl.

### Identification of wt sequence by PCR-walking

PCR-walking was optimized from [30]. Two separate tubes with 2.5 μg of genomic DNA extracted from a non-GM sample (cv. 'Pacific') were digested overnight in a volume of 100 μl with 80 U of *Eco*RV and *Sca*I (37°C). For these enzymes the absence of restriction sites in the 3' genomic flanking sequence had been previously ascertained. Enzymes were then inactivated at 65°C for 10 min and removed with chloroform; DNA was precipitated in 1/10^th ^vol 3 M NaOAc (pH 5.2) and 2 vol absolute EtOH, air dried and finally resuspended in 20 μl of sterile H_2_O. Restriction fragments were subsequently blunt-ligated with 50 pmol of double strand adapter by incubating 10 μl of the genomic digest with 10 U of T4 DNA ligase and 2 μl of its buffer in a final volume of 20 μl overnight at 16°C. The ligation was inactivated at 65°C for 10 min and the resulting "adapter library" was diluted 1:10 in TE (pH 8). For the PCR-walking reactions the sequences of adapters (5'-CTA ATA CGA CTC ACT ATA GGG CTC GAG CGG CCG CCC GGG GAG GT-3' and 5'-ACC TCC CC-NH_2_-3') and adapter primers (AP1: 5'-GGA TCC TAA TAC GAC TCA CTA TAG GGC-3' and AP2: 5'-CTA TAG GGC TCG AGC GGC-3') were slightly modified; plant genome primers (FPS1: 5'-GAT CAG AGA CAG AGA GAG AAA ATG GA-3' and FPS2: 5'-GGT GAA CTT TCT GTT GGA AGA GAA CTA-3') were specifically designed on the known sequence flanking the 3'end of the RR insert. A first PCR was performed using AP1 as forward primer and FPS1 as reverse primer to direct the amplification toward the insertion region. The 25 μl-PCR mix contained 2 μl of the adapter library, 1× Gold Buffer, 1.2 mM MgCl_2_, 0.2 mM dNTPs, 0.8 μM of each primer and 1.5 U AmpliTaq Gold^® ^DNA Polymerase (Applied Biosystems); the amplification profile consisted of 10 min at 95°C for the initial activation of the AmpliTaq Gold^® ^DNA Polymerase, followed by 30 s denaturation at 95°C, 1 min annealing at 60°C, 30 s extension at 72°C, for 35 cycles and a final extension for 10 min at 72°C. Primary PCR products were 100-fold diluted and then used as template in a nested PCR conducted in the same way as the first PCR, except that the nested primers AP2 and FPS2 were used. PCR products were run on a 1.5% agarose gel and visualized under UV light after staining in ethidium bromide solution (10 μg/ml). The bands were gel-extracted and re-amplified as above. PCR reactions were purified using the Wizard^® ^SV Gel and PCR Clean-Up System (Promega) and sequenced by MWG Biotech AG (Milan).

### End-point PCR assay

The wt allele was amplified from 100 ng DNA by PCR using primers 5'-CAA ACC ACC TTG CAC TTT GA-3' and 5'-GCG ACG ACC AGG CCA TT-3', in 25 μl of 1× Gold Buffer, 1.5 mM MgCl_2_, 0.2 mM dNTPs, 0.5 μM of each primer and 1 U AmpliTaq Gold^® ^DNA Polymerase. PCR was performed by initial 13 min at 94°C, following 40 cycles of 30 sec at 94°C, 1 min at 60°C and 2 min at 72°C, and final 7 min at 72°C. The expected wt-specific amplicon of 506 bp was visualized on a 1.8% agarose gel under UV light after staining in ethidium bromide solution (10 μg/ml).

### Real-time PCR assay

The primers (5'-AAC GGT GAA GAG AAA GAA GAG TAC CT-3' and 5'-GCG ACG ACC AGG CCA TT-3') and the TaqMan probe (5'-FAM- TGA GAT CGG GAG GGA AGA GAT TGG G -TAMRA-3') for the specific detection of the wt sequence at the RR locus were designed using the Primer Express software by Applied Biosystems. For the detection of both the construct-specific CaMVP35S-CP4-*epsps *transgenic sequence at the RR locus and the *Le1 *reference gene, primers and probes were those reported by [31]. Reactions were carried out in simplex format in 25 μl containing 200 ng DNA, 1× TaqMan^® ^Universal PCR Master mix (Applied Biosystems), 900 nM each primer and 200 nM probe. PCR conditions on an ABI Prism 7000 Sequence Detection System (SDS) from Applied Biosystems were as follows:1^st ^step: 2 min at 50°C; 2^nd ^step: 45 cycles of 15 min at 95°C and 1 min at 60°C. Amplification curves were analyzed with the ABI Prism 7000 SDS Software and for each PCR system (wt RR locus and *Le1*) Ct values were obtained.

### Validation of the quantitative real-time PCR assay

For the assessment of the suitability of the rtqPCR assay for the quantification of non-GM contaminants, standard and validation samples were used. For each level two validation samples were prepared. Three DNA extractions were performed for each sample and analyzed with three PCR replicates. For relative quantification of wt soybean in a validation sample, the normalized ΔCt values (Ct_*wt *_- Ct_*Le*1_) of calibration samples were plotted against the logarithm of the percent of wt material and the reference curve was calculated by linear regression. The normalized ΔCt values (Ct_*wt *_- Ct_*Le*1_) of validation samples were measured and used to estimate the relative amount of wt soybean by means of the regression formula. In order to determine the LOD and LOQ of the rtqPCR assay, three sets of a five-fold dilution series of 5% wt soybean DNA in RR soybean DNA were analyzed. Two hundred ng DNA of each dilution sample, corresponding approximately to 177,780 copies of the haploid genome [32], were amplified with three replications.

#### Accession numbers

Previous molecular information regarding soybean event GTS 40-3-2 is available under EMBL accessions numbers AJ308514 and AJ308515[20]. Sequences of Le1 and CaMVP35S-CP4-epsps of RR construct are submitted as EMBL accession numbers K00821 and AB209952, respectively.

## Competing interests

The authors declare that they have no competing interests.

## Authors' contributions

EB was responsible for designing the PCR assays and for carrying out all the experiments. EN was responsible for the conceptual design of this work. Both authors contributed to data analysis and to the writing of the manuscript. Both authors read and approved the final manuscript.

## Supplementary Material

Additional file 1 **Additional figure 1**. Real time quantification of adventitious presence of GMO in conventional material (conventional lot purity). Click here for file

Additional file 2 **Additional figure 2. **Real-time PCR of transgenic target obtained from a series of samples at different GMO concentrations. Click here for file

Additional file 3 **Additional figure 3.** Sub-sampling quantification of AP presence. Click here for file
