# Supplementary figures and images for: Real-time quantification of wild-type contaminants in glyphosate tolerant soybean
Source: BMC Biotechnol. 2009 Mar 6;9:16. doi: 10.1186/1472-6750-9-16 (PMC2656496; doi:10.1186/1472-6750-9-16)

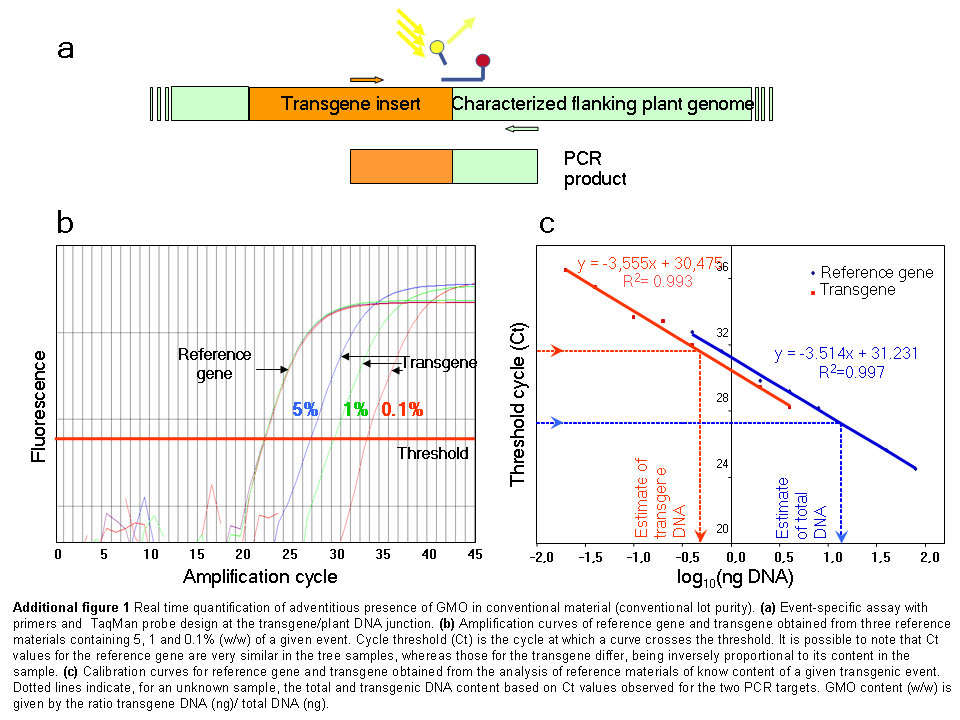

Supplement: Additional file 1 — Additional figure 1. Real time quantification of adventitious presence of GMO in conventional material (conventional lot purity). [file 1472-6750-9-16-S1.tiff]

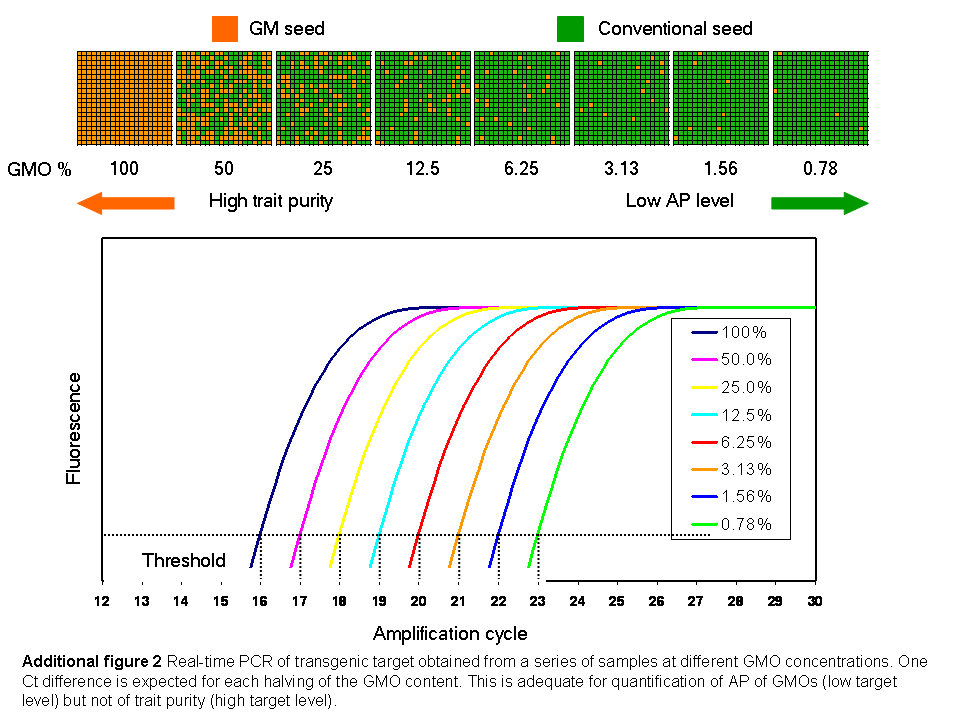

Supplement: Additional file 2 — Additional figure 2. Real-time PCR of transgenic target obtained from a series of samples at different GMO concentrations. [file 1472-6750-9-16-S2.tiff]

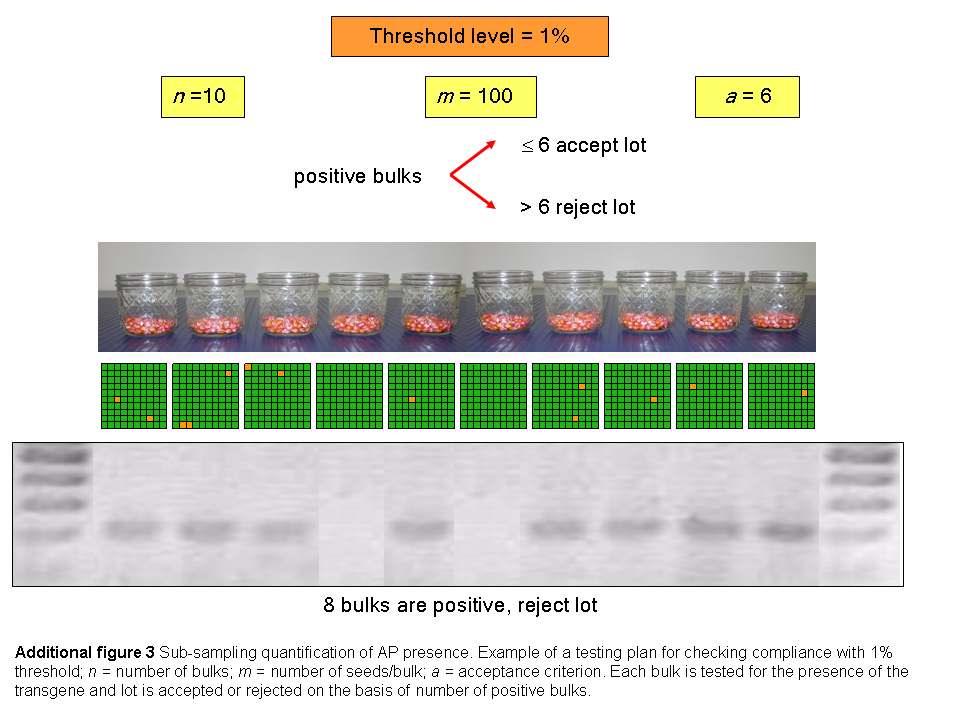

Supplement: Additional file 3 — Additional figure 3. Sub-sampling quantification of AP presence. [file 1472-6750-9-16-S3.tiff]
